# Smart Collaborative Caching for Information-Centric IoT in Fog Computing

**DOI:** 10.3390/s17112512

**Published:** 2017-11-01

**Authors:** Fei Song, Zheng-Yang Ai, Jun-Jie Li, Giovanni Pau, Mario Collotta, Ilsun You, Hong-Ke Zhang

**Affiliations:** 1 School of Electronic and Information Engineering, Beijing Jiaotong University, Beijing 100044, China; fsong@bjtu.edu.cn (F.S.); 17111017@bjtu.edu.cn (Z.-Y.A.); hkzhang@bjtu.edu.cn (H.-K.Z.); 2 Institute of Education and Economy Research, University of International Business and Economics, Beijing 100029, China; uibesafjunjie@163.com; 3 Faculty of Engineering and Architecture, Kore University of Enna, 94100 Enna, Italy; giovanni.pau@unikore.it (G.P.); mario.collotta@unikore.it (M.C.); 4 Department of Information Security Engineering, Soonchunhyang University, Asan-si 31538, Korea

**Keywords:** IoT, fog computing, ICN, smart collaborative caching, performance validation

## Abstract

The significant changes enabled by the fog computing had demonstrated that Internet of Things (IoT) urgently needs more evolutional reforms. Limited by the inflexible design philosophy; the traditional structure of a network is hard to meet the latest demands. However, Information-Centric Networking (ICN) is a promising option to bridge and cover these enormous gaps. In this paper, a Smart Collaborative Caching (SCC) scheme is established by leveraging high-level ICN principles for IoT within fog computing paradigm. The proposed solution is supposed to be utilized in resource pooling, content storing, node locating and other related situations. By investigating the available characteristics of ICN, some challenges of such combination are reviewed in depth. The details of building SCC, including basic model and advanced algorithms, are presented based on theoretical analysis and simplified examples. The validation focuses on two typical scenarios: simple status inquiry and complex content sharing. The number of clusters, packet loss probability and other parameters are also considered. The analytical results demonstrate that the performance of our scheme, regarding total packet number and average transmission latency, can outperform that of the original ones. We expect that the SCC will contribute an efficient solution to the related studies.

## 1. Introduction

The idea of the Internet of Things (IoT) has been acknowledged as a popular term for many years. Nowadays, it is attracting more attention from both industrial and academic communities than ever. Different from the Wireless Sensor Networks (WSN), IoT extends the scope of nodes and connectivities tremendously [1,2]. A batch of schemes and protocols are proposed by many standardization organizations, such as IETF, ISO, IEC, ITU, IEEE, 3GPP, GS1, to enhance the overall performance of IoT. For instance, there are several active working groups in IETF: 6Lo, 6tisch, ace, core, detnet, lwig, manet, roll and so on. This latest progress empowers IoT to be easily utilized in intelligent transportation systems [3,4], e-health [5,6], smart grid [7,8], and other common scenarios [9,10].

Fog computing is a novel paradigm which leverages many useful features of IoT to aggregate many small nodes and achieve a significant capacity. It could be perceived as an intermediate stage between cloud computing and personal computing. New research directions, such as D2D, M2M, V2V, are providing a substantial basis for it. Many new promising applications, e.g., virtual reality and augmented reality, can profit from fog computing.

However, the conjunction between IoT and fog computing is not smooth due to the drawbacks of the traditional network architecture. As a competitive clean-slate candidate, Information-Centric Networking (ICN) is engaged in modifying the original host-oriented pattern. By including more information-oriented characteristics into IoT, ICN may inspire more innovations in fog computing scenarios.

This paper focuses on several tough issues: Firstly, the connections among multiple IoT nodes are usually unconsolidated. Guaranteeing the data distributions in such circumstance is hard. However, the cooperative processing needs a firm foundation. Secondly, due to the constrained resource of IoT nodes, the information storing needs to be explored. When the capacity is not sufficient, the node should find an approach to achieve caching ability which is the basis of ICN. Thirdly, the content can be uploaded based on the requirements if previous connecting and caching capability are ready. Quickly locating the most suitable node is significant not only for the users but also for the network.

The main contributions of this work could be summarized in two aspects: (1) A new Smart Collaborative Caching (SCC) scheme is designed to enable information-centric ability in IoT, which is beneficial for better connecting the devices and providing cooperative caching in fog computing. (2) The validation results are illustrated based on analyzing the performance of common IP network, ICN flooding, ordinary SCC and enhanced SCC. The topology, scenarios, and experiments are also carefully detailed.

The basic structure of this paper is as follows: In Section 2, the related work in combining IoT with ICN and fog computing are presented respectively. In Section 3, the details of SCC design are introduced. Three necessary parts, i.e., preliminary foundation, model establishment and primary operations are involved. In Section 4, the validations are given to explore the capacity of SCC. Two scenarios, two experiments, and four candidates are implemented and discussed. In Section 5, the paper is summarized and future works are presented.

## 2. Related Work

Some recent work related with IoT in ICN and fog computing situations are presented. We believe these work could motivate more excellent achievements in the community. 

### 2.1. IoT and ICN

Among combination investigations, Lindgren et al. [11] focused on the tradeoffs of ICN in IoT structures. They also introduced the benefits the IoT gains from ICN and IoT architecture design choices to solve the application challenges. Li et al. [12] then continued their research and presented an IoT architecture based on ICN to manage the problem in combined processes. They used NDN and MobilityFirst technologies in their design and improved the service discovery part in IoT. Baccelli et al. [13] discussed the effect of utilizing ICN approaches in IoT. Some improvements on control traffic and data path management are illustrated. Moreover, they also showed a comparison of CCN with other IoT standards. In [14], the authors performed an analysis and implemented several ways for the application of ICN in IoT scenarios. They demonstrated the possibility of this combination based on many different ICN performance facts. Waltari and Kangasharju [15] introduced an approach of using CCN in IoT environments, which can solve the Internet challenges brought by the increasing number of access devices. A forwarding strategy of data transmission is proposed in [16] by Melvix et al. It focused on reducing the energy consumption in an IoT system. The authors also modified the naming scheme, and their approach has a better performance than IP-based techniques. Datta and Bonnet [17] proposed a unified naming scheme for interests to facilitate the interaction between two regions in NDN based IoT architecture.

For information processing, Siris et al. [18] investigated the overhead of data messages in the ICN-based IoT. They designed a simulation of three optimization models in an ICN testbed with a PSI prototype and showed their performance in reducing traffic loads. Borgia et al. [19] introduced a framework which combines ICN with global cloud via mobile facilities at the edge of the network. They aim to offer better data management and service provisioning for IoT. Amadeo et al. [20] presented a structure of ICN usage in an IoT environment. The proposed naming scheme can help with the data exchange between the devices in the network. Ahlgren et al. [21] proposed an approach for managing the sensor data from IoT instruments with CCN technique. In [22], Quevedo et al. discussed the benefits of applying CCN in an IoT architecture. They studied several caching mechanisms in consideration of bandwidth and energy consumption. Hail et al. [23] presented a new caching strategy called pCASTING. They used ndnSIM to execute a simulation and evaluated the performance based on the energy efficiency of the network. Ye et al. [24] proposed a novel scheme for data processing by utilizing NDN in reconfiguring the relevant logic in network. The evaluation showed that it could reduce the energy cost during the transmission and save bandwidth.

For communication enhancement, Chen et al. [25] presented the benefits of using MobilityFirst in an IoT scenario to support the communication. This paper also explained the feasibility of using other special ICN architectures. Dong et al. [26] presented an ICN-based distributed Resource Directory (RD) scheme for IoT which takes the consideration of request forwarding (pushing) and name resolution. It aim to significantly reduce the routing table size and the network overhead. Hahm et al. [27] introduced their work by combining ICN with a particular IoT approach, TSCH. This combination may improve the energy efficiency and decrease the need for error management in the MAC layer. In [28], Dong and Wang proposed a context-based scheme for IoT communication in an ICN architecture which ensure the correct information forwarding with the arrangement of FIB and PIT. They also compared it with the existing methods to show its performance. Hail and Fischer [29] presented an IoT application AAL, which is able to offer better health service for the elderly. This framework realizes a timely communication among different devices by using a NDN structure. Moreover, they continued to present a new NDN-based API [30] for IoT service which also focused on developing the communication service. Amadeo et al. [31] studied the reliable data forwarding in IoT traffic whose background has adopted NDN. They introduced three solutions and discussed their feasibility and shortcomings, especially their performance in reducing the overhead and network load.

### 2.2. IoT and Fog Computing

In combination investigations, Sarkar and Misra [32] presented a mathematical formulation for fog computing which is used to support for IoT application. They compared the performance of fog computing in energy consumption and service latency with existing mechanisms. Yannuzzi et al. [33] discussed the challenges faced by modern computing approaches in the IoT and introduced the reasons why a mixed fog computing scheme is more suitable for IoT environments. Giang et al. [34] analyzed the feasibility of applying fog computing in IoT. They introduced a new programming method called DDF which combines cloud and fog from service perspective. The simulation showed the better performance in different IoT applications. Lee et al. [35] focused on the particular security issues brought by fog computing in IoT scenarios. They also evaluated the present security mechanisms of IoT clouds. Sarkar et al. [36] proposed the comparison of the fog computing with the traditional cloud computing. Moreover, they demonstrated the suitability of fog computing for its better performance in dealing with the requirements from a large number of IoT applications. Bibani et al. [37] presented a demo based on PaaS for enabling IoT applications in hybrid cloud and fog environment, which reduces latency and promotes processing performance. Salman et al. [38] discussed several novel approaches about their application in IoT architecture and highlighted the benefits of MEC for the IoT which has adopted the fog computing technique.

For resource arrangement, a model about the agreement of resources was proposed in [39]. The authors aimed to realize an efficient and reasonable resources management, and they executed the evaluation work on the CloudSim toolkit to observe the performance of the model. Skarlat et al. [40] presented a framework to deal with the disadvantages of fog computing in the release of resources. Their design reduces the delay in the processing and has a shorter transmission time. Aazam et al. [41] focused on the topic of the resource evaluation in consideration of the Relinquish Rate (RR). Their experiments were carried out in an actual IoT environment and demonstrated the efficiency in reducing the waste of resources for their mechanism. They also proposed an approach [42] whose management of the resources is based on the type and the price of services. This framework can deal with the complicated requirements of the IoT-based services caused by their fast increasing number. Abedin et al. [43] proposed a model which utilizes fog computing to realize a more efficient communication among the devices in the IoT. Their model used a conception of matching theory to realize node pairing. Aazam and Huh [44] analyzed the feasibility in the combination of fog computing and smart gateways. Their design has taken into account of many types of delay to enhance the performance of service provisioning in the IoT. Taneja and Davy [45] described an algorithm for determining the locations of the analytic data operators which can be used in a fog framework.

For data integration, Jalali et al. [46] focused on the consumption of energy in different IoT applications, and they proposed a method which facilitates the integration of fog computing and micro grids. It can help with the storage and computation of the applications. Rauniyar et al. [47] discussed the security problems in the IoT. They presented a fog computing framework and data offloading scheme to detect and deal with disasters. In [48], Chiang and Zhang presented a conception called Fog Servers. It is utilized in smart city scenarios to offer high-quality service based on the measure of energy. Moreover, the experimental results show that adaptive FSs have a better performance due to the dynamic power consumption and capacity. Hu et al. [49] built a face identification system based on the fog computing technology. It is important for the security applications in the IoT. Dastjerdi and Buyya [50] discussed the benefits offered by fog computing in IoT regarding how to deal with the considerable amount of data which is generated from the applications. The authors deem that the fog computing can effectively connect the cyber and physical environment. Prazeres and Serrano presented a novel framework SOFT-IoT in [51]. They focused on the data processing to realize a reasonable management of the service delivery operation for the subordinate systems in IoT architectures. Gia et al. [52] proposed a monitor system based on fog computing with the aim of data management in healthcare. This system combines with smart gateways to offer better detection results.

### 2.3. Comparison and Discussion

For IoT+ICN, the current combinations did not fully consider the requirements of fog computing, which will seriously limit the scalability of these schemes. As the key functionalities, achievements in information processing and communication enhancement only focus on high-level improvements. Stable connection establishment is still a tough issue. For IoT+Fog, the existing combinations mainly investigate the macroscopic and generic topics, which is quite necessary in its early stage. However, most researches in resource arrangement and data integration are still based on a device-centric concept. Information itself cannot be smartly exchanged among massive nodes.

Previous literature reviews help us to rethink the construction method of a comprehensive caching solution. There are already some initial attempts in this area. More specifically, Abdullahi et al. [53] introduced a conceptual framework for embedding ICN into ubiquitous computing paradigms as an API. Although this paper included many relevant elements and illustrated complicated layer structures, the detail of the particular scheme were not provided. Khan et al. [54] integrated “Centrality” into ICN and proposed a novel concept named Content-Based Centrality (CBC). The authors also designed a placement algorithm for content and validated the performance. Nevertheless, this paper only focused on the distribution approach for content, which means the content finding approach is still open. Motivated by the same target, Khan et al. [55] switched the application scenario to a mobile environment. By merging the spatial temporal features of content and the eligibility of nodes, the author proposed a new caching scheme. To balance the cost and available capacity, a coalition game is adopted to encourage the contribution of nodes. Although this work considered multiple key perspectives, an important issue, i.e., joining and leaving of nodes, is still lacking.

## 3. Smart Collaborative Caching for IoT

To achieve information-centric IoT targets, the content should be appropriately cached inside multiple intermediate nodes and easily found by the network. Such a difference will greatly enable more possibilities in fog computing. Therefore, an efficient caching scheme is essential. By analyzing the basic demands of SCC, the preliminary foundation is presented. Then, specific steps of establishing the model are given based on a widely approved algorithm. To further support different scenarios and improve overall performance, four primary operations are also investigated.

### 3.1. Preliminary Foundation

Driven by the requirements of SCC, our scheme is established based on a test of time solution: Chord [56]. We first introduce the shared concepts. Then the details of SCC will be proposed by considering the typical characteristics of an information-centric IoT. Relevant algorithms will also be presented. Only one operation was supported in the Chord protocol: *Map a key onto a node.* Let us assume that the key identifier is a fixed length “abbreviation” of original key (a file, a web page, a name, and so on). The node identifier is a fixed length “abbreviation” of a network node (a router, a switcher, a server, and so on). By using a hash function, one could easily map the original key into an *m*-bit key identifier. The associated network node information like IP address, MAC address, serial number, and so on, could be selected as the hash function input to generate the *m*-bit node identifier. For simplification, the term “key” (“node”) will refer to both the original key (network node) and key (node) identifier under the hash function. *Consistent hashing* is adopted to ensure each node will be in charge of roughly the same number of keys, which is useful not only for averaging the burden of nodes, but also for reducing large-scale key migration. Chord further enhance the scalability of consistent hashing by maintaining a small number of routing items in each node. If there are *N* nodes in the network, both the information of other nodes and lookup message of one request are *O*(log *N*). *Identifier circle, successor, and predecessor* are three significant elements in Chord. Both the key and node are sharing the same identifier circle space. All identifiers are anticlockwise placed in a circle based on their value modulo 2*m*. Key *k* will be assigned to the first node whose identifier is equal to or bigger than *k*. This node is named successor node of key *k*, expressed by a successor (*k*). If the key belongs to 0 to 2*m* − 1, actually the successor (*k*) is just the first node clockwise from *k*. The first node whose identifier is smaller than *k* is named predecessor node of key *k*, expressed by predecessor (*k*). Similarly, the predecessor (*k*) is just the first node anticlockwise from *k*. The *Finger Table* is an essential element stored in each node. It is a particular routing table which contains *m* items at most. Each item records a mapping relationship between one interval and one successor node who is in charge of this range. The interval is the circular range separated by multiple break points. As long as a specific node *n* is provided, the identifier of each break point could be easily calculated based on:

(1) $$\left(n+2^{k-1}\right)\;\mathrm{mod}\;2^{m},1\leq k\leq m$$

Then the interval range will be generated automatically. Since not all the slots in a circle can be filled with nodes, one node might be mapped to multiple intervals.

### 3.2. Model Establishment

**Figure 1:**
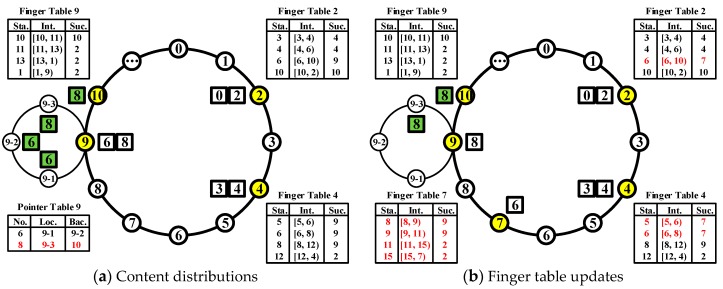
A schematic of SCC with clusters and content.

In the information-centric IoT, the situation is not as simple as before. Here are two practical examples: when a node is supposed to cache the original key, the capacity is not big enough; when a node leaves the system without sending the notification, there will be no original key which was cached in this node. Therefore, “map a key onto a node” is not appropriate anymore in such scenarios. According to the target of ICN, the original key should be stored in the entire network, not just in a single node. Our basic idea is to separate the traditional binding between the key identifier and the original key. The former will be handled by the leader of IoT cluster (introduced in Figure 1), the latter will be stored by the leader itself or members insider cluster. It is also possible to cache the latter in other clusters. Then all clusters should establish a “Pointer Table” to maintain the mapping between the key identifier and the original key. However, the additional complexity must be well controlled. These collaborative operations are crucial for SCC, which enable it to be a strong tool in fog computing.

A schematic circle of SCC is constructed in Figure 1a. For simplification, the term “content” (“cluster”) will refer to both the original content (IoT cluster) and content (cluster) identifier under the hash function. The distinction is easy to find based on context. Each cluster should maintain a constant identifier which is hashed from the information of cluster leader, cluster member, or other related components. It should not be changed until the last member has left the network. The cluster leader can represent the whole cluster and mark itself with the cluster identifier. Assuming *m* is equal to 4, there will be 16 empty slots (characterized by the circles with white) for both clusters and contents. The position of slots is not proportionally spaced to better illustrate the main part. Four existing clusters represented by their cluster leaders (marked by the circles with yellow) are 2, 4, 9 and 10, respectively. The cluster members (characterized by the thin circles with white), i.e., 9-1, 9-2 and 9-3, should have a direct connection with cluster leader 9. The circle linked them is not another Chord circle. The identifiers of cluster members are just the extensions of cluster leaders, which means it is only visible inside cluster by the local members. The six content (marked by the squares with white) are 0, 2, 3, 4, 6 and 8, respectively. Three finger tables for cluster 2, 4 and 9 are given to locate the cluster that is in charge of the content. The content 6 and 8 (marked by the squares with green) are cached in 9-1, 9-2, 9-3 and 10 due to the limited capacity of cluster leader 9. It is suggested that the backup content of a cluster leader should be stored in its successors. They could be easily found even if this cluster is disappeared. A pointer table of cluster leader 9 is also given to identify the mapping between the content and its location.

### 3.3. Primary Operations

Four main actions of SCC will be discussed. More details are provided during the demonstration process:

#### 3.3.1. Caching and Finding 

To present the whole process of content caching and finding, we choose a specific content 8 as the main object.

Assuming cluster 2 had received an original content and its hash function result is 8, the search inside cluster 2 will be initiated immediately. Based on the partition rule, the successor of content 8 should be located. Since there are *m* items in the finger table, an efficient method is required. “Sta.”, “Int.” and “Suc.” are abbreviations for start, interval, and successor. The simple idea is to check the items one by one. However, it will take three steps to find interval [6, 10). Intuitively, it only needs two steps if one checks from the end. We need to investigate if the step number of this scheme is always smaller than that of the previous one when large *m* is adopted. According to Formula (1), the theory behind that is it will halve the distance to the target after each item checking. The more clusters IoT has, the fewer steps it will need. Since a cluster knows more information about their neighbors than others do, it makes sense to check it successor before the reverse lookup. When cluster 9 had been found, a caching request of content 8 will be sent from cluster 2. Due to the limited capacity of cluster leader 9, the caching task of content 8 will be assigned to cluster member 9-3 and cluster 10, respectively. It is also acceptable to execute fragmentation for content 8, but such operation is not recommended due to the integrality considerations. A new item should be added into pointer table 9 (marked by the red font). “Loc.” and “Bac.” are abbreviations for location and backup.

**Figure 2:**
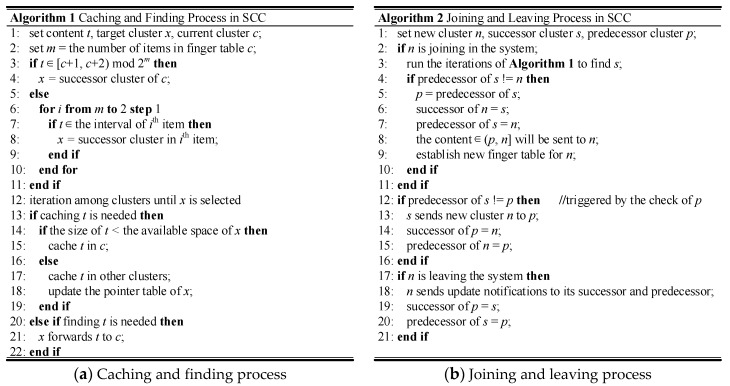
Algorithms used in SCC.

Assuming a request for content 8 was generated from cluster 4, finding processes will utilize the same idea to locate the target cluster. When cluster 4 checks the first item of its finger table, it will not succeed because the content 8 does not belong to the interval [5, 6). Based on the previously discussed algorithm, cluster 4 should check the last item. Although it will fail again, the next step will successfully find the interval [8, 12) as well as the successor cluster 9. Then a request for content 8 will be sent from cluster 4 directly. Since the cluster leader 9 does not cache this content, it will check the pointer table to find the practical location of content 8. We only use an ergodic algorithm here as a primitive version. More efficient lookup algorithms could be designed based on that. When cluster member 9-3 and cluster 10 are found, the content 8 will be sent to cluster 4. The relevant pseudocode is illustrated in Figure 2a.

#### 3.3.2. Joining and Leaving

We mainly focus on the cluster level changes since the fluctuations insider cluster could be handled by the cluster leader and members.

Assuming cluster 7 wants to join in the system via cluster 4, it is acceptable to choose an arbitrary cluster. For the changes of successor and predecessor, since cluster 4 had a macroscopic understanding of the whole network, regarding how many clusters are online, cluster 7 could realize that its position should be in the middle of existing cluster 4 and 9. Then the cluster 7 will modify its successor to cluster 9 and send an update notification to it. If the operation is successful, cluster 9 will mark cluster 7 as the predecessor and forward the relevant content (i.e., content 6) back. Based on the “Stabilization” actions of Chord, each node needs to check its successor periodically to ensure the correctness. When cluster 4 sends the check request to cluster 9, it will be notified that cluster 9 is not the predecessor anymore and cluster 7 is the new one. Then the cluster 4 will modify its successor to cluster 7 and send an update notification to it. If the operation is successful, cluster 7 will mark cluster 4 as the predecessor. For the changes to finger tables, there are two ways to construct the finger table of cluster 7: Ask its introducer cluster 4 or copy from its next neighbor cluster 9. The updates for all other nodes could be executed simultaneously. The result of cluster 7 join is presented in Figure 1b. All the changes in finger tables had been marked with red color.

Assuming cluster 7 wants to leave the system, the necessary procedures are quite similar with that of joining. When cluster 9 receives an update notification from cluster 7, it will change the predecessor from cluster 7 to cluster 4. The relevant content (i.e., content 6) should be sent back to cluster 9 as well. Cluster 7 may send an update notification to cluster 4 to trigger a quick change of the successor. However, even these actions are not executed by cluster 7, the performance of the whole system will not be affected seriously. Due to the success of its applications, more and more recent papers [57,58] are presented to enhance this protocol. We believe all these works are also beneficial for SCC. The relevant pseudocode is illustrated in Figure 2b.

## 4. Validation and Discussion

**Figure 3:**
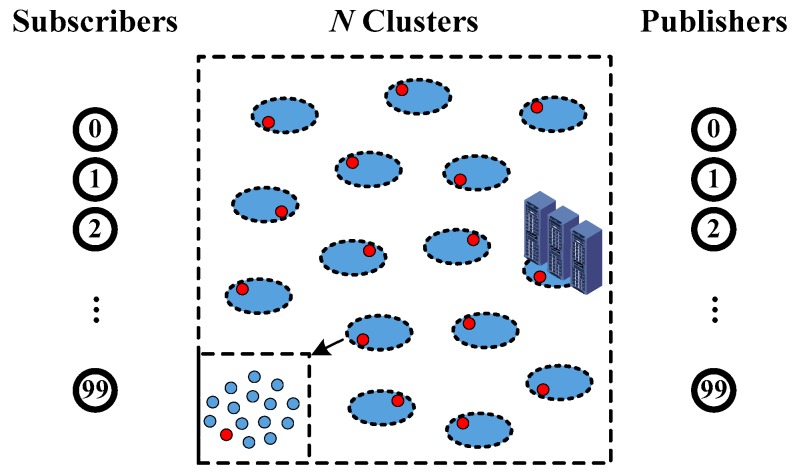
Topology establishment for SCC validation.

A topology (shown in Figure 3) is carefully built to compare and analyze the overall performance of different schemes. Multiple IoT nodes are gathered to form a cluster, which is the basic element of fog computing. A cluster is composed of a leader (marked by the circles with red) and a group of members (marked by the circles with blue). The enlarged view of a cluster is illustrated at the bottom left corner. There are totally *N* clusters (located in the center area), 100 subscribers and 100 publishers (located at the left and right sides, respectively) in the network. The clusters are numbered from 1 to *N*, according to the distance between the cluster and subscriber (*N* means the farthest cluster). A rendezvous server with aggregation function belongs to one cluster that is near to the publishers. Let us assume that each cluster has caching ability to store a complete packet and one message will not be separated into multiple packets. The period interval of requests is stable.

Four kinds of schemes are enabled inside multiple clusters between subscribers and publishers. The first one is a traditional unicast scheme in the IP network. The second one is a flooding scheme in ICN, which spread the packets to all neighbors. The third one is our SCC scheme, which aims to significantly reduce the number of packets and the transmission latency. The last one is ICN SCC-E, which is an enhanced version of SCC based on one hop searching [59]. It could maintain one hop feature during the cluster searching process by storing more items in their finger tables.

Two scenarios (simple status inquiry and complex content sharing) are established, and two experiments (calculating packet number and transmission latency during the requests and content exchanging) are executed by considering four candidates. In the first experiment, the subscribers will generate and send requests. The best case and the worst case, regarding overall packet number, will be calculated respectively. Then the average number of them are marked with different signs in figures. In the second experiment, similar to the first one, we calculate the average transmission latency between sending the requests and receiving the content.

### 4.1. Scenario One: Simple Status Inquiry (One Request and One Response)

Suppose each subscriber requests one piece of content from 100 publishers. The corresponding applications might be data collections, remote control, etc. All the information sent by the publishers can be compressed into one packet inside the rendezvous server. We first carried out a theoretical analysis for multiple candidates. Both the packet number and transmission latency will be checked carefully.

For the packet number, when the subscribers want to achieve contents, the best case is: the first subscriber initiates one request to the cluster 1. If the packet can reach the rendezvous server through one hop, it is the shortest path. Then the rendezvous server sends one request packet to each publishers. When receiving these requests, the publishers should return 100 responses back to the rendezvous server. Since all these responses will be aggregated into a content packet and returned back to cluster 1, only one packet appears in the network. Finally, this packet is forwarded to subscriber 1. During previous procedures, there are 1 + 1 + 100 + 100 + 1 + 1 packets in total, i.e., *P_1B_* = 204 (*P* for “Packet number”, 1 for “the first subscriber” and *B* for “Best”). The worst case is: after the subscriber sends the request, such packet reaches the rendezvous server through *N*-1 clusters, which is the longest path. In this case, *N* − 1 packets will be forwarded. Then 100 request packets are transmitted from the rendezvous server to 100 publishers. Since the reverse direction of data flow will generate the same packet, the total packet number *P*_1*W*_ (*W* for “Worst”) can be calculated via Equation (2):

(2) $$P_{1W}=1+\left(N-1\right)+100+100+\left(N-1\right)+1=200+2N$$

When the *i*th subscriber initiates the request (*i* ≠ 1), since the request of previous subscriber(s) had been received, the aggregation of required content should be stored inside the rendezvous server. Therefore, the interaction between rendezvous server and publishers can be waived. To minimize the number of packets in the network, the path should be as short as possible. The best case is: it takes one hop to reach the rendezvous server and the required content returns back to the subscriber in the same way. During such procedures, there are 1 + 1 + 1 + 1 packets in total, i.e., *P_iB_* = 4. The worst case is: it takes *N* − 1 hops in both forward and backward directions inside network. The total packet number *P_iW_* should be equal to 2*N*. To illustrate the mean level of all cases, we calculate their value range by using Equation (3):

(3) $$\left[\overset{100}{\underset{i=1}{\sum }}P_{iB}/100,\overset{100}{\underset{i=1}{\sum }}P_{iW}/100\right]=\left[6,2+2N\right]$$

For the transmission latency, it is defined as the period between request sending and content receiving. All the Round Trip Times (RTTs) are equivalently set and the fluctuations of jitters are set to 5 ms. We assume that the delay of each link is half of *RTT* and the 100 publishers receive (send) the requests (responses) simultaneously. In forward direction, when the first subscriber initiates a request, it has to pass by several clusters and the rendezvous server before arriving at the publishers. The minimum latency is 3 × *RTT*/2, i.e., only 1 cluster is involved. In backward direction, when all publishers have returned 100 responses, the aggregation delay inside the rendezvous server can be ignored since the *RTT* is relative large. If the reverse path is maintained, the overall minimum latency *L*_1*B*_ (*L* for “Latency”) should be 3 × *RTT*. Similar with previous analysis, the maximum latency will appear if all clusters are involved in both directions. The calculation of *L*_1*W*_ is shown in Equation (4):

(4) $$L_{1W}=\frac{RTT}{2}\times \left(1+\left(N-1\right)+1+1+\left(N-1\right)+1\right)=RTT\times \left(1+N\right)$$

When the *i*th subscriber initiates the request (*i* ≠ 1), it will take 1 (*N* − 1) hop(s) between the first cluster and the rendezvous server in the best (worst) case. Therefore, the *L_iB_* and *L_iW_*, in terms of minimum and maximum delay, for each round are 2 × *RTT* and *N × RTT*, respectively. The value range of latency can be obtained according to Equation (5). Both the packet number and transmission latency calculation process of the other candidates can follow the same methodology.

(5) $$\left[\overset{100}{\underset{i=1}{\sum }}L_{iB}/100,\overset{100}{\underset{i=1}{\sum }}L_{iW}/100\right]=\left[\frac{201RTT}{100},\frac{\left(1+100N\right)RTT}{100}\right]$$

**Figure 4:**
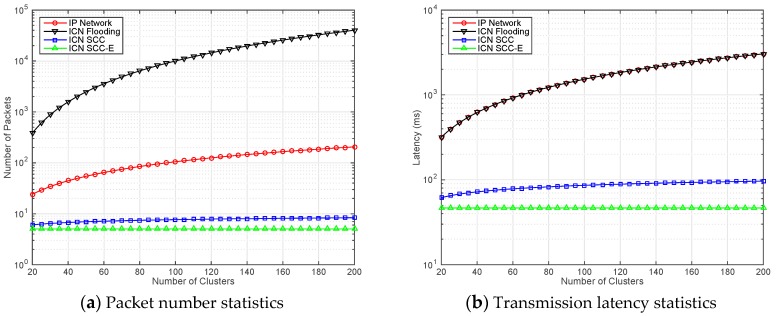
Results for scenario one: simple status inquiry.

In Figure 4a, the packet number statistics for different schemes are illustrated. The horizontal axis is set to the number of clusters in the network. To better highlight the difference, we adopt logarithm for vertical axis display. The curve with black (at the top of the figure) demonstrate the packet number in ICN Flooding scheme. Although content caching is enabled in all clusters, plenty of unnecessary packets are still generated and forwarded inside whole network. It is significant to design an appropriate policy even ICN is adopted in IoT scenarios. The curves with red and blue (at the middle area of the figure) represent the IP Network and ICN SCC, respectively. As we expected, with the increasing of network scale (in terms of cluster number), the traffic volume of traditional IP network is getting bigger. The growth rate in this scheme is not as high as that of ICN Flooding. However, it still brings some additional burdens for the clusters. For ICN SCC scheme, the caching cost and searching efficiency are properly balanced. The growth rate of packet number is obviously controlled. The curve with green (at the bottom of the figure) shows that the fluctuation of ICN SCC-E is always stable. It proves that one hop searching is effective in SCC and the clusters can find the content with limited cost.

In Figure 4b, the transmission latency statistics for different schemes are illustrated. From top to down, the gaps between ICN Flooding and IP network are quite small. Due to the similarity of macro procedures in these two schemes, the advantages of ICN is not fully demonstrated. However, using SCC and SCC-E to replace flooding scheme can respectively reduce the latency 80.7% and 85.4% if 20 clusters exist in current network. With the increasing of clusters, such performance enhancement is more obvious. For a particular case, when the number of clusters *N* is changed from 80 to 140, we record the fluctuations in the vertical axis. The growth rate of ICN Flooding and IP Network curves is 74%. The values in ICN SCC curve grow 10.2%, which is quite slow comparing with previous two schemes. The values in ICN SCC-E curve are invariant.

### 4.2. Scenario Two: Complex Content Sharing (One Request and Multiple Responses).

Suppose each subscriber requests 100 pieces of content from one publisher. The corresponding applications might be video surveillance, file transmission, etc. To better reflect the real situations, we consider drop events in this scenario. Set the packet loss probability of each link among clusters is *p* (0 < *p* < 0.5). Let us assume that the request packets, retransmitted packets and packets in last hop will not be dropped by the network.

Before calculating the packet number and transmission latency in four candidates, a fundamental issue must be analyzed in depth: Will longer path or shorter path generate larger packet number during the transmissions? It is straightforward in previous scenario because no packet is lost. To answer such question quantitatively, we need to consider a generic case. The topology is minimized by adding one subscriber, one cluster, one rendezvous server (located inside another cluster) and one publisher. For the backward path, the number of data packets *X* (for the shorter path case) can be achieved based on Equation (6):

(6) X=100+100(1−p)+100(1−p)

where each item is for each independent link. Then the topology can be enlarged by adding *N*-2 more intermediate clusters. For the backward path, the number of data packets *Y* (for the longer path case) can be recalculated based on Equation (7):

(7) $$Y=100+\left[100\left(1-p\right)+\cdots +100{\left(1-p\right)}^{N-1}\right]+100{\left(1-p\right)}^{N-1}$$

where the items inside square brackets are for the links among clusters. If the target condition is “larger packet number belongs to longer path”, the value of *Y* should be bigger than that of *X*. Based on the geometric progression theory, such condition could be equivalently converted to Formula (8):

(8) $$\frac{\left(1-p\right)-{\left(1-p\right)}^{N-1}+p{\left(1-p\right)}^{N-2}}{p}>1$$

**Figure 5:**
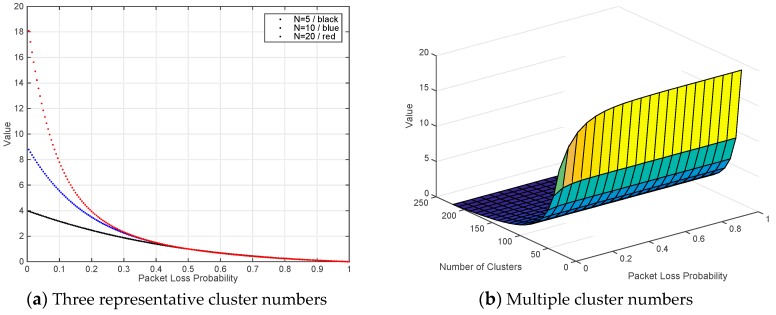
Relationship analysis for *p*, *N* and packet number.

To further explore the relationship between *p*, *N* and packet number, we draw two analytical figures in Matlab. Figure 5a illustrates the results of three representative *N* (5, 10 and 20 are represented with black, blue and red, respectively). With the increasing of packet loss probability (from 0 to 1), all curves are getting down. The critical value appears when *p* is around 0.5, i.e., the value of polynomial will approach 1. Since more values of *N* will lead to serious overlaps, we only reserve three curves in Figure 5a. More details are demonstrated in Figure 5b from the three-dimensional perspective. It also proves that 0 < *p* < 0.5 is a reasonable selection for scenario two.

**Figure 6:**
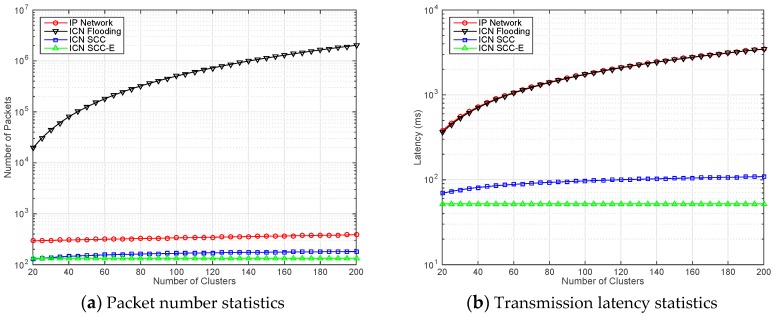
Results for scenario two: complex content sharing.

Figure 6a illustrates the packet number statistics. Although the sequence (from top to down) of four curves are the same with Figure 4a, the values of many points have been changed. We still select 80 and 140 as two representatives for *N*. The increasing rates for ICN Flooding and IP Network curves are 207.8% and 9.3%, respectively. The disadvantages of the flooding scheme are also displayed. By adding new features of ICN SCC, the packet number growth is only 7.4%. Such a benefit is more obvious if the ICN SCC-E scheme is utilized.

Figure 6b illustrates the transmission latency statistics. The variations in scenario switching and experiment adjustment did not drastically influence the validation results. The performance enhancement of ICN SCC and ICN SCC-E is confirmed.

## 5. Conclusions

Fog computing has provided unprecedented and attractive offers in multiple perspectives. IoT is a significant and promising candidate to provide a response for that. By analyzing the advanced characteristics of ICN, a Smart Collaborative Caching (SCC) scheme has been proposed to assist an information-oriented IoT establishment. We presented the details of cluster composition, content processing, position management, and so on. Four representative SCC actions, i.e., caching, finding, joining and leaving, have been carefully introduced. The relevant algorithms have also been presented. A validation topology including 100 subscribers, 100 publishers and *N* clusters was established to compare the performance differences among four solutions (IP network, ICN flooding, ICN SCC and ICN SCC-E). Two scenarios (based on the number of responses) and two experiments (based on packet number and transmission latency) have been designed. The results demonstrated that naïve flooding in ICN is almost useless for an IP network improvement. Both ICN SCC and ICN SCC-E could achieve acceptable efficiency in all experiments. In the future, we would like to investigate the applicability of SCC in more scenarios and further optimize the data exchange processes. We believe that SCC could have an essential role in the information-centric IoT and promote the relevant developments in fog computing.
